# Importance of Arsenic and pesticides in epidemic chronic kidney disease in Sri Lanka

**DOI:** 10.1186/1471-2369-15-124

**Published:** 2014-07-28

**Authors:** Channa Jayasumana, Ranil Gajanayake, Sisira Siribaddana

**Affiliations:** 1 Department of Pharmacology, Faculty of Medicine and Allied Sciences (FMAS), Rajarata University of Sri Lanka (RUSL), Mihintale, Sri Lanka; 2 Nephrology Unit, Yale New Haven Hospital, New Haven, CT, USA; 3 Department of Medicine, Faculty of Medicine and Allied Sciences (FMAS), Rajarata University of Sri Lanka (RUSL), Mihintale, Sri Lanka

## Abstract

In a recent study published by the National Project team on chronic kidney diseases of unknown origin in Sri Lanka, we believe there to be flaws in the design, analysis, and conclusions, which should be discussed further. The authors wanted to emphasis Cadmium as the major risk factor for chronic kidney disease of unknown etiology in Sri Lanka while undermining the importance of Arsenic and nephrotoxic pesticides. To arrive at predetermined conclusions the authors appear have changed and misinterpreted their own results. The enormous pressure applied by the agrochemical industry on this issue may be a factor. Herein, we discuss these issues in greater detail.

## Background

Since early 1990s, chronic kidney disease was reported among paddy farmers in the North Central Province of Sri Lanka. Since then it has spread to other agricultural communities in the dry zone. This has not been attributed to any of the known causes of CKD like diabetes, hypertension, glomerulonephritis etc. Thus, it was named as chronic kidney disease of unknown origin (CKDu). The growing number of patients with CKDu places burden on health resources due to high cost of dialysis and transplantation. Many stakeholders have worked to explore the etiologies and treat these patients. We read with interest the paper published by the National Research Project Team for CKDu, Sri Lanka [1]. However, we believe there were flaws in the design, interpretation and conclusion of this study.

## Discussion

The objectives of the study were to determine the prevalence and identify the risk factors for CKDu by comparing cases and controls.

Authors have used multi-stage cluster sampling and calculated the district-wise prevalence of CKDu in endemic areas; prevalence figures for Anuradhapura district was 15.1%, Polonnaruwa 20.6% and Badulla 22.9%. These prevalence figures were calculated by selecting six Divisional Secretariat (DS) areas randomly from these three districts and then randomly selecting 22 Grama Niladhari Divisions (GND) from the six selected DS areas. Prevalence was calculated from 0.23% sample from the total population. Selection of the primary cluster (DS) or secondary cluster (GND) is not clear. Sampling interval not calculated. There are 44 DS areas and 1216 GND areas in these three districts with a wide population range (see Table 1). Authors should have used probability proportionate to size (PPS) method. This means that a cluster with large population will have a greater chance of being picked than a cluster with smaller population. This will assure that the sample is representative. PPS produces a sample that is self-weighted, and as the cluster size is also constant (100) each household will have an equal probability of being selected.

**Table 1:** **Three endemic districts and administrative divisions with population**[2]

**District**	**Population**	**No. of DS areas**	**Population range of DS**	**No. of GND areas**	**Population range of GND**
Anuradhapura	856,232	22	22,227–69,590	557	113–6,013
Polonnaruwa	403,335	7	36,424–82,138	292	126–5,223
Badulla	811,758	15	19,540–100,434	567	148–4,315

From each household (2200, there is a zero missing in the flow chart) all inhabitants between 15–70 years (6698) were invited to take part in the research and 74% (4958) consented. Family history of kidney disease was 20% among patients, and familial clustering was not considered in the sample size calculation.

CKDu is geo-environmental disease and not uniformly distributed [3]. It is more common in areas where people use hard water for drinking but not all areas with hardness in ground water is affected [4]. When there is surface water from irrigation systems and pipe-borne water that tastes better people use that for drinking. Also there should be heavy use of agrochemicals in the endemic areas for 20 years. So selecting some DS areas may give skewed prevalence figures.

Was there significant difference between 26% who refused to participate and participants? It will be informative to know the basic demographic characteristics of the people who have refused to take part.

In the study, investigators have selected Hambanthota to recruit control participants from non-endemic area. Hambanthota is a district located in South-East Sri Lanka with a long coastline unlike other CKDu endemic regions. Further, the cultivation pattern and crops are different from that in Anuradhapura or Polonnaruwa [5]. How the control participants from Hambanthota are selected is not mentioned in the text or in the flow chart negating a basic premise in a case control study. If the controls are not selected in a representative manner the calculation of odds ratio in case control study is not valid.

The text and the flow chart describe patients and participants with a history of snakebite were excluded from the study. However, exact figures are not given. A previous study has shown 12% adult population in the endemic region and 16% of patients with CKDu have history of snakebite [6]. Also 37% patients with acute kidney injury (AKI) after snakebite develop CKD [7]. These wide exclusion criteria may have potential bias towards underestimating the prevalence of CKDu. Why patients with no AKI following snake bite was excluded? If we accept this case definition, why a control group with CKD of known causes not studied?

The authors focused on Arsenic (As), Cadmium (Cd) and pesticide residues. Arsenic is not a well-known nephrotoxin. The possible role of As in CKDu in Sri Lanka was first pointed out by the CJ (first author) at the scientific committee meeting of the National research program for CKDu held on 18^th^ January 2011 (Additional file 1). However, it has not been acknowledged anywhere in the paper.

Same findings that are published by Jayathilake et al. were submitted to the Ministry of Health Sri Lanka by the project team as the final public report of the “Investigation and Evaluation of Chronic Kidney Disease of Uncertain Aetiology in Sri Lanka” on January 2013 [8]. Ministry of Agriculture appointed an expert committee (CJ was a member) on CKDu and the said committee based its policy decisions on this report. The same report mentioned arsenic excretion in urine was significantly higher in healthy participants in the endemic area (mean 92.443, median 36.99, min 0.02, max 966.29 μg/g creatinine) compared to those living in the controlled area (mean 56.572, median 42.025, min 5.38, max 350.28 μg/g creatinine) (*p* < 0.001). Urinary As excretion in CKDu patients was significantly lower (mean 45.477, median 26.3,min 0.4, max 616.6 μg/g) compared with urinary As excretion in normal participants in the endemic area (mean 92.443, median 36.99, min 0.02, max 966.29 μg/g creatinine) (*p* < 0.01). These are presented in the Table three of the paper, but under the subheading ‘arsenic, cadmium, lead and other elements in urine’ (Page 5) the authors say “There was no significant difference in urine arsenic and lead concentrations in CKDu cases compared to controls”. This is false. Also they have not mentioned the significant differences between urine arsenic values between the endemic and non-endemic area in the footnote of the Table three or anywhere in the text. This was clearly mentioned in the final public report. However, they mention about the significant difference between urine Cd between patients, controls from endemic area and controls from non-endemic area. Is it a mistake or have they conveniently forgotten?

In the page 10 second paragraph says that mean urine concentration of As in CKDu cases was above levels known to cause oxidative injury to the kidney (20.74 μg/g creatinine). The mean urinary concentrations of As is well above, not only in CKDu cases (45.477 μg/g creatinine) but also from healthy participants from endemic (92.443 μg/g creatinine) and non-endemic areas (56.572 μg/g creatinine). They have even quoted a Taiwanese study describing linear dose response relationship between As and kidney disease. Then they discuss about nephrotoxicity due to co-exposure of cadmium and arsenic again without discussing about nephrotoxicity of As.

A Bangladesh study shows the blood Selenium level is inversely associated with urinary As level in people exposed to As as selenium is a well documented anti-oxidant [9]. Jayatilake et al. have shown 63% of the tested CKDu patients had low serum selenium values.

Interestingly, in the initial submission of this paper, it is mentioned “chronic exposure to low levels of Cd through food chain coupled with a deficiency of selenium and concurrent exposure to arsenic and pesticides may play a role in the pathogenesis of CKDu in Sri Lanka” [10]. However, in the final published version of the paper this statement was missing even though reviewers have not mentioned anything related to arsenic or this statement.

Low excretion of As in urine and accumulation in nails and hair of patients with advanced stages CKDu is seen [1]. Unusually, urine Cd excretion is high in patients with advanced CKDu when compared with unaffected participants. Also these patients have high Cd levels in hair and nail similar to As. However, tobacco chewing and smoking was not assessed as a confounding factor when measuring the Cd content [11]. Patients with CKDu are excreting more Cd, while high amount of it was found in the nail and hair samples with compared to controls in the same area. Therefore, urinary Cd data presented in the paper is against the common reasoning and contradict the data on lead (Pb) and As. Further, the paper concluded that there is a dose effect relationship between the concentration of Cd in urine and the stage of CKDu. There are several studies showing that there is no increase in urine Cd levels in patients with CKDu in Sri Lanka [3,12]. A Swedish study shows that urinary Cd excretion is an unreliable indicator of renal damage and can overestimate the risk of renal toxicity from low level Cd exposure [13]. Temporary changes in urine flow or normal physiological variability can cause high urine Cd excretion.

There is another facet in this argument. Cd is mainly present in the fertilizer, *Triple Super Phosphate* given as a part of agriculture subsidy by the government [14]. Arsenic in addition is present in the imported pesticides available in the open market [15]. Is this the real reason about discussing nephrotoxicity of Cd extensively and discussing flimsily about As in the discussion? Cd is a well-known nephrotoxic but not As. Has the authors taken easy way out?

Authors have mentioned that they analyzed urine samples of 57 CKDu cases and 39 controls from non-endemic area for pesticide residues. Nevertheless, the results are given only in CKDu cases. At the same time, they conclude people in the endemic area are chronically exposed to pesticides. What about the pesticide exposure in non-endemic Hambanthota, a predominantly agricultural district?

In the other hand if known nephrotoxic pesticides or its residues are present above the reference level in urine of patients with CKDu, finding out the origin of it was crucial. The drinking water of patients with CKDu is free or minimally contaminated with As, Cd and Pb. The postulate presence of these heavy metals is from the food chain. This can give the wrong message that water in the endemic area is safe. Several researchers have already shown that water in the endemic areas is heavily contaminated with pesticides and its residues [16]. There was public debate in the newspapers and in the electronic media about the As and heavy metal contamination in agrochemicals polluting the drinking water and the environment. Some members in the National Research Project Team for CKDu mentioned in the acknowledgement section of the paper participated in this debate.

## Conclusion

This debate over the quality of agrochemicals available in Sri Lanka has influenced the results and conclusion of the study. Findings related to As and pesticides are concealed, and prominence is given to Cd.

## Response

By Shanthi Mendis

Email: mendiss@who.int

Address: Senior Adviser Noncommunicable Diseases, World Health Organization, Geneva, Switzerland

Jayasumana et al. [17], in their correspondence, make an unfounded allegation that the debate over the quality of agrochemicals available in Sri Lanka has influenced the results and conclusion of the Ministry of Health/World Health Organization study [1], and that the findings related to arsenic and pesticides are concealed, and prominence is given to cadmium. This is absolutely untrue.

The Ministry of Health/World Health Organization study did not only focus on cadmium, in a biased manner. The potential role, if any, of aluminium, arsenic, calcium, chromium, copper, magnesium, potassium, selenium, sodium, strontium, titanium, zinc and pesticides were investigated. In the initial submission of the paper it is stated that ‘chronic exposure to low levels of cadmium through the food chain coupled with a deficiency of selenium and concurrent exposure to arsenic and pesticides may play a role in the pathogenesis of CKDu in Sri Lanka’. Subsequently, at the request of reviewers, further dose response analyses were performed for arsenic, cadmium and lead. As the results in the final publication clearly demonstrate [1], while there is a dose effect relationship between urine cadmium and the stage of CKDu, there is no significant dose–effect relationship between urine arsenic and the stage of CKDu. As stated in the conclusions of the final publication [1], our results indicate chronic exposure of people in the endemic area to low levels of cadmium, lead and arsenic through the food chain and pesticides. Significantly higher urinary excretion of cadmium in individuals with CKDu, coupled with the dose–effect relationship between urine cadmium levels and CKDu stages, indicated that cadmium is a risk factor for the pathogenesis of CKDu in Sri Lanka.

Jayasumana et al. [17], also claim that snake bite should not be an exclusion criteria in the case definition of CKDu. We do not agree, because a significant number of snake bite victims develop chronic kidney disease following snake envenomation [7].

Although the problem of CKDu has been recognized in Sri Lanka since the late 1990, when our population prevalence study was initiated, there was only one published study that reported a CKDu prevalence of 2–3% [3]. To avoid further delay in assessing the magnitude of this public health problem, a prevalence study was commenced on a representative sample in one district, using World Health Organization funds. Later, more funding was secured from the National Science Foundation of Sri Lanka for a comprehensive project and the prevalence study was expanded to two other districts. Many different sampling methods and designs are available for research studies. One of the main reasons for conducting a prevalence study is to plan health services for those who are affected. Using multistage cluster sampling the age-standardised prevalence of CKDu was 12.9% (95% confidence interval [CI] = 11.5% to 14.4%) in males and 16.9% (95% CI = 15.5% to 18.3%) in females [1]. As CKDu is a life threatening condition, the priority, should be to plan health services for the affected, using the available prevalence estimates, while further research continues.

It is regrettable that Jayasumana et al. [17] accuse us of publishing predetermined conclusions and misinterpreting results due to pressure applied by the agrochemical industry, without any evidence. At no stage during the research project was such pressure applied by any entity. Our findings are based on the results of painstaking and rigorous research conducted over a four year period and not on any predetermined conclusions. The unfounded accusations of Jayasumana et al. [17] do not invalidate any of the results of the most comprehensive research study up-to-date on the subject of CKDu in Sri Lanka [1]. Our results call for urgent public health measures [18], to safeguard the health of the population in the endemic areas, including regulatory control of fertilizer and agrochemicals. It is encouraging that the inter-ministerial committee appointed by the President of Sri Lanka has already taken steps to implement some of these measures [19-21].

## Competing interests

The authors declare that they have no competing interests.

## Authors’ contributions

CJ and RG wrote the initial manuscript. SS added new points and improved the presentation. All authors read and approved the final manuscript.

## Authors’ information

CJ (MBBS) is a lecturer in the Department of Pharmacology, Faculty of Medicine and Allied Sciences (FMAS), Rajarata University of Sri Lanka (RUSL). Further he is the director, National program for prevention of kidney diseases under the Ministry of special projects, Sri Lanka.

RG (MBBS, MD) is working in the Nephrology unit, Yale New Haven Hospital, Connecticut, USA.

SS (MBBS, MD, FCCP, FRCP_Edin_) is Professor of Medicine and Chair, Department of Medicine, and Dean of the FMAS, RUSL.

## Pre-publication history

The pre-publication history for this paper can be accessed here:

http://www.biomedcentral.com/1471-2369/15/124/prepub

## Supplementary Material

Additional file 1 **Minutes of the scientific committee meeting of the National research program for CKDu, Sri Lanka held on 18**^**th **^**January 2011.** Click here for file
