# Supplementary material for: Importance of Arsenic and pesticides in epidemic chronic kidney disease in Sri Lanka
Source: BMC Nephrol. 2014 Jul 28;15:124. doi: 10.1186/1471-2369-15-124 (PMC4119246; doi:10.1186/1471-2369-15-124)
Supplement: Additional file 1 — Minutes of the scientific committee meeting of the National research program for CKDu, Sri Lanka held on 18 th January 2011. [file 1471-2369-15-124-S1.pdf]

දුරකථන) 2698475
தொலைபேசி) 2698490
Telephone) 2698507

ෆැක්ස්) 2692913
பெக்ஸ்) 2694860
Fax)

විද්‍යුත් තැපැල)
மின்னஞ்சல் முகவரி) postmaster@health.gov.lk
e-mail)

වෙබ් අඩවිය)
இணையத்தளம்) www.health.gov.lk
website)

සුවසිරිපාය
சுவசிரிபாய
SUWASIRIPAYA

මගේ අංකය)
எனது இல)
My No.)

ඔබේ අංකය)
உமது இல)
Your No. :)

දිනය)
திகதி) 02/02/2011
Date)

සෞඛ්‍ය අමාත්‍යාංශය
சுகாதார அமைச்சு
Ministry of Health

Minutes, Scientific Committee Meeting

National Research Programme for CKDu

Minutes of the Scientific Committee Meeting of the National Research Programme for CKDu, held on January 18, 2011 has been attached herewith for your kind perusal and necessary follow-up.

We thank all the participants for the active participation and contribution at above meeting.

Dr. P. G. Maheepala
Chair, Scientific Committee (CKDu)
Additional Secretary (Medical Services)
Ministry of Health

385, පූජ්‍ය බද්දේගම විමලවංශ හිමි මාවත, කොළඹ 10, 385, வணக்கத்துக்குரிய பத்தேகம விமலவங்ச தேரோ மாவத்தை, கொழும்பு 10.
385, Rev. Baddegama Wimalawansa Thero Mawatha, Colombo 10, Sri Lanka.

National Research Programme on CKDu

Minutes of the Scientific Committee Meeting held on January 18, 2011

The Scientific Committee Meeting of the National Research Programme on Chronic Kidney Disease of Unknown Aetiology was held on January 18, 2011 at the Epidemiology Unit Auditorium. The objectives of this meeting were as follows:

- To review the current status of research activities under the National Research Programme
- To review the new study proposals submitted by research groups
- To get briefs on other research activities on CKD
- To discuss the way forward

Members of the Scientific Committee comprised of researchers, academics, physicians and administrators and representatives from the Ministry of Health and World Health Organization participated at this meeting (list of participants in annexure).

Dr. Ananda Amarasighe, Consultant Epidemiologist welcomed the participants on the behalf of Ministry of Health. Dr. Firdosi Rustom Mehta, Country Representative, WHO in his opening remarks briefed about the role of WHO in the research programme. WHO will continue to facilitate resource mobilization efforts and technical collaborations. WHO has already provided USD 300,000 and he thanked the National Science Foundation for their contribution of USD 700,000 (through the Treasury). He also mentioned about the national level symposium planned to be held in late April or May this year.

There were three sessions at the meeting:

1. **Briefs on research activities under the National Programme**
   The following presentations were made in the 1st session:
   - Population Prevalence Study by Dr. Samith Ginege, Epidemiology Unit
   - GPS Mapping by Mr. Dilip Hensman, WHO, Country Office Sri Lanka
   - Postmortem Study by Dr. Upul Karunaratne, Postmortem Study Group
   - Other Studies by Dr. N. Janakan, Epidemiology Unit

2. **New study proposals for review**
   The following presentations were made in the 2nd session:
   - Effect of cyanotoxins in the pathogenesis of CKDu by Dr. Dhammika Dissanayake, University of Peradeniya
   - Socioeconomic impact of CKD by Prof. Saroj Jayasinghe, University of Colombo

3. **Briefs on other studies related to CKDu**
   The following presentations were made in the 3rd session:
   - Cyanobacteria, Cyanotoxins & CKDu by Dr. Dhammika Magana-Arachchi, Institute of Fundamental Studies
   - Health implications (CKDu) of fluoride in ground water of the dry zone by Mr. J. P. Padmasiri, Institute of Fundamental Studies
   - Interstitial nephritis in rats given bore well water from North Central Province by Dr. Mayuri Thammitiyagodage, Medical Research Institute
   - Arsenic, Mercury & CKDu by Dr. Channa Jayasumana, university of Rajarata

Summary of discussion and the way forward

Salient points & discussion	Action	Responsibility
Study groups have agreed to share their GIS data among them.	GIS work done by WHO & others will be shared once the accuracy is ensured	DH/WHO & study groups
Postmortem study group agreed to speed-up the sample collection. Consultant JMO, Anuradhapura mentioned that because of the stringent selection criteria for the control group, they are facing difficulties in completing the required number of control group postmortems.	Explore the possibility of relaxing the selection criteria for controls<br>Finish the postmortem sample collection at least by the 1st week of February and send samples to University of Antwerp, Belgium	NJ/WHO<br><br>Upul<br>SH/WHO
Prof Ileperuma mentioned that despite the decision taken at the 1st SC meeting, no postmortem samples have been provided to him for analysis of aluminum, fluoride & other metals	To discuss with Antwerp about sharing samples collected already or to extend the postmortem study further to collect more samples	NJ/WHO<br>Upul<br>SH/WHO
SC suggested to include control areas in the NCP itself (identified low prevalent areas) in addition to already selected control areas (Hambantota) for environmental sampling	To discuss further with the environmental study group & Dr. SM/WHO HQ	NJ/WHO
There were 2 presentations on cyanobacteria & CKDu. Presentations were followed by extensive discussion with inputs from experts in the field.	To form a study group on "cyanobacteria & CKDu" and share the expertise & lab capacity for the future studies	Study Group<br>NJ/WHO
Feasibility of sharing the socioeconomic data available with the 'population prevalence study group' with the 'socioeconomic impact study group' was discussed.	To share the relevant data once a formal request is made by the relevant study group	DH/WHO<br>Study Group
Prof Saroj Jayasinghe proposed forming a core group of researchers & meet more frequently (other than the scientific committee meetings) to coordinate the research programme	To discuss with the study groups	Addl. S/MoH<br>WR

Some of the other issues discussed:

- Need for finding suitable early disease markers was discussed. As CKDu is a chronic disease, early diagnosis would help in stopping the disease progression. However, at the field level for screening programmes/ prevalence studies use of advanced early disease markers is not feasible and hence urine ACR has been used as a surrogate indicator for renal impairment. Consultant Nephrologist, TH Anuradhapura mentioned for clinical follow-up of patients also urine ACR is a useful investigation.
- As the University of Rajarata is located in the North Central Province, it was agreed to have collaborations with the research group there in the ongoing and future research activities.
- Need for studying the socioeconomic impact of CKD was reiterated by the WR. Report of this study can be effectively used as an advocacy document to solicit the support of policy makers and the political leadership for mitigation and future prevention activities.
- Considering the human suffering in the high risk areas and the time taken for the research effort, suitability of a public health message on the need for safe drinking water was discussed. Access to safe drinking water is a fundamental right. However, adequacy of scientific evidence for such a message directly linking drinking water and CKDu was queried by some of the participants.
- Dr. Maheepala stressed the need for the individual researchers and groups to work together under the national research effort. He also requested them not to share hypotheses/ findings to the media and public without complete scientific evidence. The research effort should be based on proper research methodology. Further, quality control of analytical data is vital and it should be ensured with the assistance of local and international accredited centres.
